# Effects of Occupational Therapy Interventions on Occupational Performance and Physical and Cognitive Fatigue in Adults with Multiple Sclerosis: A Systematic Review of Randomized Controlled Trials

**DOI:** 10.3390/jcm15208060

**Published:** 2026-10-17

**Authors:** Edgar Vásquez-Carrasco, Braulio Henrique Magnani Branco, Roberto Lopez-Andaur, Jordan Hernandez-Martinez, Sebastián Burgos-Carrasco, Laura Ochoa-Martinez, Cristian Sandoval-Vásquez, Francisca Peña, Pablo Valdés-Badilla

**Affiliations:** 1 School of Occupational Therapy, Faculty of Psychology, Universidad de Talca, Talca 3465548, Chile; edgar.vasquez@utalca.cl; 2 Centro de Investigación en Ciencias Cognitivas, Facultad de Psicología, Universidad de Talca, Talca 3465548, Chile; 3 VITALIS Longevity Center, Universidad de Talca, Talca 3465548, Chile; 4 Program in Health Promotion, Cesumar University (UniCesumar), Maringá 87050-900, Brazil; braulio.branco@unicesumar.edu.br; 5 Exercise and Rehabilitation Sciences Institute, School of Occupational Therapy, Faculty of Rehabilitation Sciences, Universidad Andres Bello, Santiago 8370035, Chile; r.lopezandaur@uandresbello.edu; 6 Department of Physical Activity Sciences, Universidad de Los Lagos, Osorno 5290000, Chile; jordan.hernandez@ulagos.cl; 7 Department of Education, Faculty of Humanities, Universidad de La Serena, La Serena 1700000, Chile; 8 Académico Escuela de Terapia Ocupacional, Facultad de Salud y Ciencias Sociales, Universidad de Las Américas, Concepción 4030000, Chile; sburgos@udla.cl; 9 Facultad de Salud, Escuela de Tecnología Médica, Universidad Santo Tomás, Los Carreras 753, Osorno 5310431, Chile; lochoa@santotomas.cl; 10 Carrera de Terapia Ocupacional, Facultad de Ciencias de la Salud, Universidad Autónoma de Chile, Temuco 4810101, Chile; 11 Departamento de Medicina Interna, Facultad de Medicina, Universidad de La Frontera, Temuco 4811230, Chile; 12 Department of Physical Activity Sciences, Faculty of Education Sciences, Universidad Católica del Maule, Talca 3465548, Chile; 13 Sports Coach Career, Faculty of Life Sciences, Universidad Viña del Mar, Viña del Mar 2520000, Chile

**Keywords:** adults, neurodegenerative diseases, occupational performance, physical activity, rehabilitation

## Abstract

**Background/Objectives:** Multiple sclerosis (MS) frequently limits participation in daily occupations and is associated with disabling fatigue. However, the effects of occupational therapy (OT) remain uncertain because available trials have evaluated heterogeneous interventions and outcomes. This systematic review synthesized evidence from randomized controlled trials (RCTs) on occupational performance and physical and cognitive fatigue in adults with MS. **Materials and Methods:** Seven bibliographic databases were searched from inception to 10 August 2026: MEDLINE via PubMed, Scopus, the Cochrane Central Register of Controlled Trials (CENTRAL) via the Cochrane Library, Web of Science Core Collection, APA PsycINFO via EBSCOhost, CINAHL Complete via EBSCOhost, and ProQuest Central. Two independent reviewers performed study selection, data extraction, and risk-of-bias assessment using RoB 2. Certainty of evidence was evaluated using GRADE. Given the clinical and methodological heterogeneity, findings were synthesized narratively following the Synthesis Without Meta-Analysis guideline. The protocol was registered in PROSPERO (CRD420251115448). **Results:** Seven RCTs involving 488 randomized participants were included. Findings for occupational performance were inconsistent: one small waitlist-controlled trial reported large short-term benefits from occupational performance coaching, whereas an active-comparator trial found improvements in both groups without clear superiority. Other findings showed no comparative benefit or provided only indirect evidence. Fatigue findings were similarly inconsistent: one 20-participant pilot trial favored a sleep-targeted OT intervention, two trials found no comparative benefit, and one large trial transiently favored usual-care OT. The certainty of evidence was very low for both outcomes. **Conclusions:** The available evidence does not consistently establish that OT interventions improve occupational performance or reduce physical and cognitive fatigue in adults with MS. Larger, adequately powered RCTs using clearly defined OT interventions, standardized outcomes, and longer follow-up periods are required.

## 1. Introduction

Multiple sclerosis (MS) is a chronic immune-mediated disorder of the central nervous system that affects approximately 2.8 million people worldwide and represents a major cause of non-traumatic disability among young and middle-aged adults [1,2,3]. Its heterogeneous motor, sensory, cognitive, emotional, and autonomic manifestations can progressively restrict independence, employment, social participation, and health-related quality of life [1,4,5,6]. Although disease-modifying therapies primarily target inflammatory activity and disease progression, rehabilitation remains essential for addressing persistent limitations in daily functioning, activity, and participation [4,7,8].

Fatigue is among the most prevalent and disabling symptoms of MS and may involve physical, cognitive, and psychosocial dimensions [8,9,10]. Its effects frequently interact with mobility limitations, upper-limb dysfunction, cognitive difficulties, sleep disturbances, and environmental barriers, thereby compromising the performance of meaningful daily activities [9,10,11]. Occupational performance refers to a person’s ability to select, organize, and satisfactorily perform occupations within a particular physical, social, and cultural context [12]. Accordingly, perceived performance, satisfaction, independence, and participation constitute clinically relevant outcomes when evaluating rehabilitation interventions for adults with MS [9,11,12].

Occupational therapy (OT) is a person-centred health field that uses occupation-based and goal-directed interventions to promote participation in meaningful activities and social roles [11,12]. OT interventions for adults with MS may include fatigue and energy-management education, self-management programmes, cognitive and metacognitive strategies, upper-limb rehabilitation, environmental modification, assistive technology, and occupational performance coaching [8,11,13,14]. Technology-assisted approaches, including virtual reality, are also being increasingly incorporated into rehabilitation to provide interactive and task-oriented practice [15,16,17]. However, recent reviews demonstrate substantial variability in the technologies employed, intervention content, outcome measures, follow-up periods, and certainty of the resulting evidence [15,16,17]. Technology-assisted and multidisciplinary interventions should therefore be distinguished from direct OT interventions when the independent contribution of the occupational therapist cannot be isolated [8,16,17].

Previous scoping reviews and clinical practice guidelines have mapped a broad range of OT interventions for adults with MS but have also identified considerable methodological heterogeneity and variable certainty across outcomes [11,13,14]. The 2026 Cochrane review and the present review address complementary rather than identical questions. Both concern adults with MS; however, the Cochrane review evaluated OT across daily functioning, participation, health-related quality of life, and adverse effects, included randomized and non-randomized controlled studies, and searched the literature through November 2024 [8]. In contrast, the present review was restricted to RCTs reporting occupational performance or physical and cognitive fatigue, extended the search through to 10 August 2026, and used a SWiM-guided narrative synthesis because the intervention contrasts and outcome formats were not sufficiently comparable. In addition, the evidence was classified as direct, comparative, adjunctive, embedded, or indirect, allowing each finding to be interpreted according to whether an independent OT effect could reasonably be identified. Thus, this review provides an outcome-focused and attribution-sensitive complement to the broader Cochrane synthesis rather than a replication of it.

Against this background, a focused synthesis of randomized evidence examining self-perceived occupational performance and physical and cognitive fatigue was considered necessary [8,11,13,14]. Therefore, this systematic review aimed to synthesize evidence from randomized controlled trials concerning the effects of OT interventions and rehabilitation approaches relevant to OT practice on occupational performance and physical and cognitive fatigue in adults with MS. Interventions were interpreted according to whether OT was delivered directly, used as an adjunct or comparator, or embedded within a broader multidisciplinary programme, and findings were synthesized without meta-analysis because of the clinical and methodological heterogeneity.

## 2. Materials and Methods

### 2.1. Protocol and Registration

This systematic review was conducted in accordance with the Cochrane Collaboration guidelines [18], and reported following the PRISMA 2020 statement, including both the checklist and flow diagram (Supplementary Table S1) [19]. The protocol was prospectively registered in the PROSPERO database (CRD420251115448).

### 2.2. Eligibility Criteria

We included peer-reviewed individual or cluster RCTs published in any language through to 10 August 2026. Participants were adults aged ≥18 years with clinically diagnosed MS, without restrictions on phenotype, severity, disease duration, clinical status, or setting. Eligible trials evaluated interventions classified as discussed in Table 1, lasted ≥3 weeks, included a randomized comparator, and reported baseline and post-intervention data on occupational performance, related activity or participation outcomes, or physical or cognitive fatigue. Mixed samples were eligible only when data for adults with MS were extractable. Non-randomized studies and grey literature were excluded. Eligibility was defined using the PICOS framework (Table 1) [18].

### 2.3. Operational Definition of Occupational Therapy Interventions

An intervention was considered directly delivered occupational therapy when it was explicitly identified as such and was delivered or supervised by an occupational therapist, or structured according to a recognized occupational therapy model. Its content was required to address engagement or performance in meaningful occupations through occupation-based activities, individualized goal setting, coaching, self-management, fatigue management, environmental adaptation, or assistive technology [8,11,12]. The minimum duration of three weeks was applied only as an exposure criterion.

To avoid over-attribution, studies were classified as direct or comparative occupational therapy, adjunctive evidence when both groups received conventional occupational therapy, embedded evidence when occupational therapy formed part of a multidisciplinary programme, or indirect evidence when no occupational therapy intervention was delivered. Only direct and comparative studies were interpreted as evidence of occupational therapy effectiveness; adjunctive, embedded, and indirect findings were reported separately because the independent contribution of occupational therapy could not be established [8,11].

### 2.4. Information Search Process and Database

Seven bibliographic databases were searched from inception to 10 August 2026: MEDLINE via PubMed, Scopus, the Cochrane Central Register of Controlled Trials (CENTRAL) via the Cochrane Library, Web of Science Core Collection, APA PsycINFO via EBSCOhost, CINAHL Complete via EBSCOhost, and ProQuest Central. Controlled vocabulary and free-text terms related to multiple sclerosis, occupational therapy, and adults were combined and adapted to the syntax and searchable fields of each database. No restrictions on language or publication date were applied. Grey literature was not searched. The complete database-specific search strategies, platforms, search dates, searchable fields, limits, and numbers of records retrieved are provided in Supplementary Material S1. The final search also included checks for errata or retractions affecting the included studies.

### 2.5. Study Selection Process and Data Collection

All studies were exported to Mendeley Reference Manager (version 2.116.2), and the selection process is depicted in the PRISMA flow diagram. Two authors independently conducted the searches and systematically screened titles, abstracts, and full texts. Duplicates were removed after completing the search of all databases, and no discrepancies were identified at this stage. Potentially eligible articles were then re-evaluated in detail, with exclusions documented based on predefined eligibility criteria. A third reviewer independently audited the entire data selection and extraction process to reach a consensus among the authors.

### 2.6. Data Synthesis

Data from the included studies were extracted into a standardized form using Microsoft Excel^®^ (version 16.81), following Cochrane guidelines [20]. Two researchers independently performed the data extraction and compared their results to ensure accuracy. A third reviewer provided oversight. Extracted variables included authors, country, study design, sample size, group sizes (n), mean age (years), type of intervention and control, training volume (frequency, duration, intensity), assessments of occupational performance and physical and cognitive fatigue, and main outcomes.

### 2.7. Risk of Bias

The risk of bias in the included RCTs was assessed using the RoB 2 tool [21]. Two reviewers performed the initial assessment, which was subsequently reviewed by additional authors. Any discrepancies were resolved through discussion until a consensus was reached.

### 2.8. Certainty of Evidence

The certainty of the evidence was appraised following the GRADE approach [22], classifying findings as high, moderate, low, or very low. Initial ratings started at high certainty given the inclusion of RCTs but were downgraded when issues such as risk of bias, inconsistency, imprecision, lack of transparency, or potential publication bias were identified. Independent evaluations were performed by two reviewers, and any discrepancies were settled through discussion with a third reviewer.

## 3. Results

### 3.1. Study Selection

**Figure 1:**
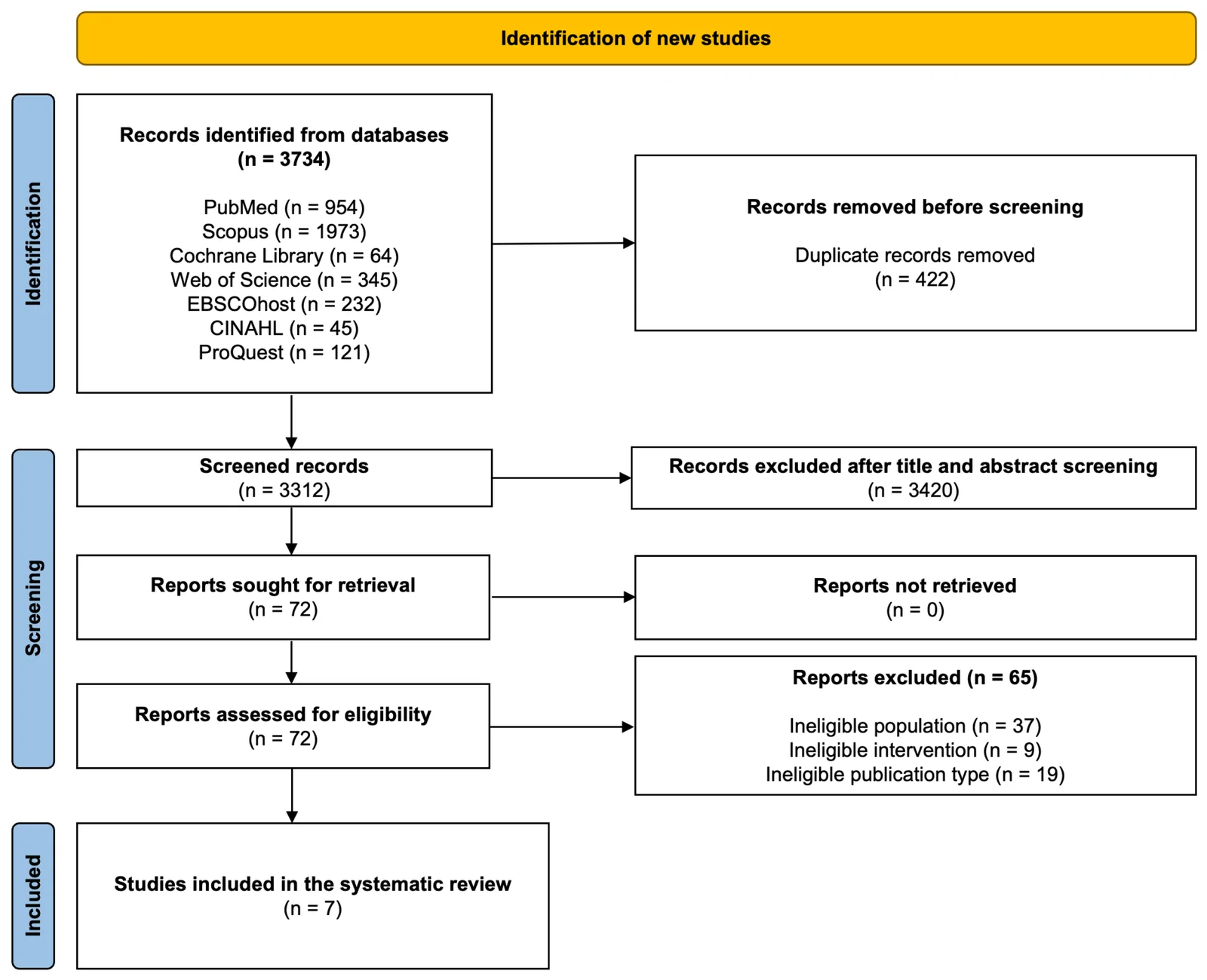
Flowchart of the systematic review selection process.

The database searches identified 3734 records. After removing 422 duplicates, 3312 records underwent title and abstract screening, of which 3240 were excluded. All 72 reports sought for retrieval were obtained and assessed for eligibility. Of these, 65 were excluded because of an ineligible population (n = 37), an ineligible intervention (n = 9), or an ineligible publication type (n = 19). Consequently, seven studies met the eligibility criteria and were included in the systematic review [23,24,25,26,27,28,29] (Figure 1).

### 3.2. Risk of Bias

**Figure 2:**
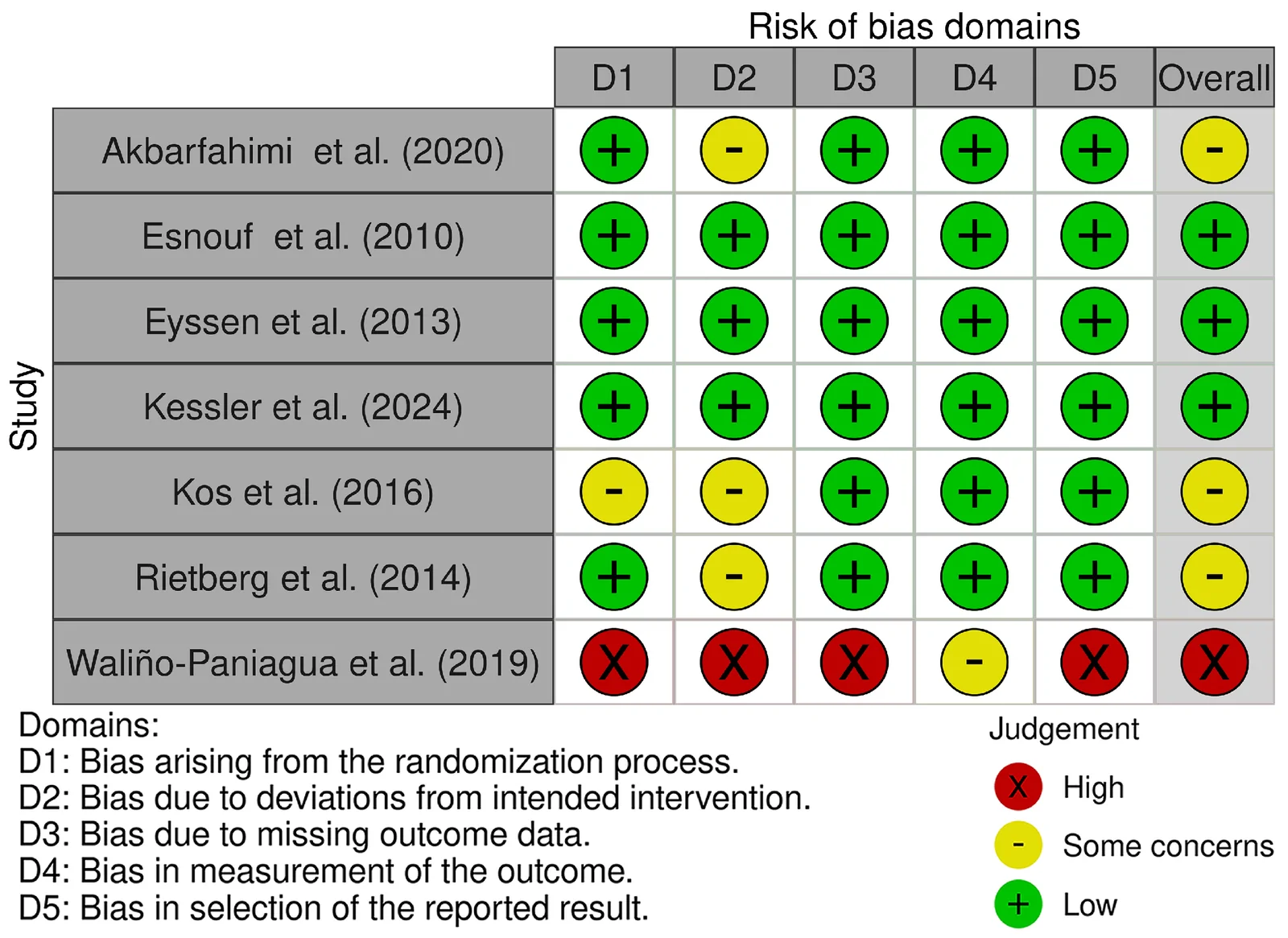
Summary of the risk of bias assessments [22,23,24,25,26,27,28].

**Figure 3:**
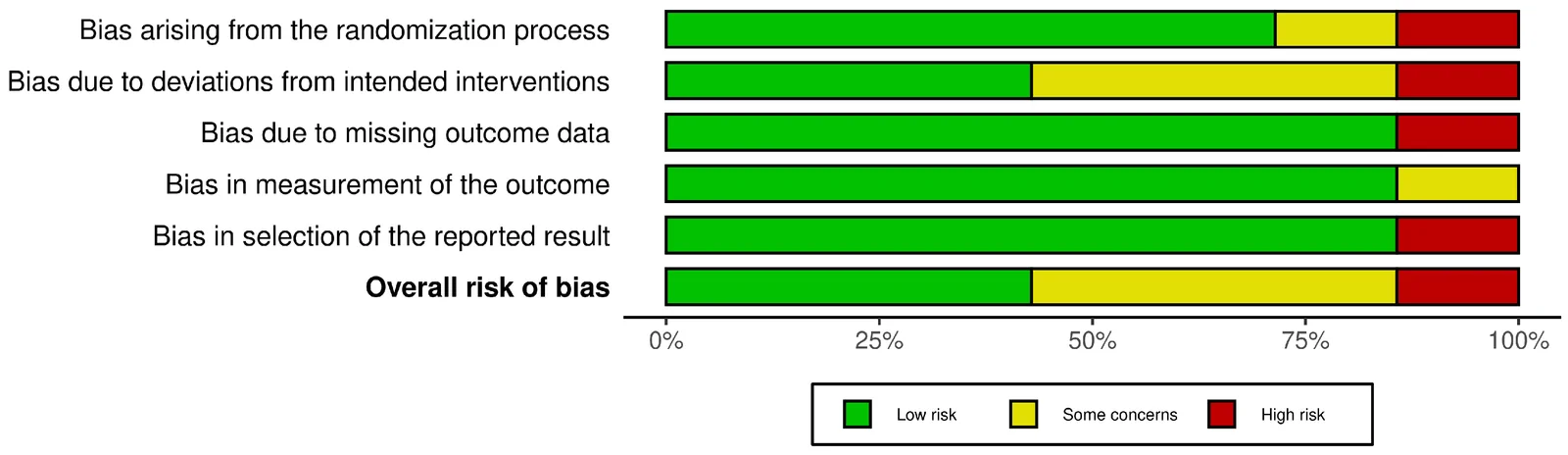
Risk of bias: traffic light graph.

Three studies were judged to have a low overall risk of bias [23,26,27], three presented some concerns [25,28,29], and one was judged to have a high risk of bias [24]. The high-risk judgment was mainly related to substantial attrition and limitations in the randomization and outcome-reporting processes. Concerns in the other studies were principally associated with missing outcome data, deviations from the intended interventions, and insufficient information regarding prespecified analyses. The domain-level judgments are presented in Figure 2 and Figure 3. These assessments were considered when interpreting the direction and certainty of the findings.

### 3.3. Characteristics of the Studies

The seven included studies were conducted in Iran, the United Kingdom, the Netherlands, Canada, Belgium, and Spain [23,24,25,26,27,28,29]. Six were individually randomized controlled trials, whereas Eyssen et al. used a multicentre cluster-randomized design [26].

The interventions and comparators differed substantially. Three studies evaluated a directly delivered OT intervention against care as usual, a waitlist, or an active comparator [25,27,28]. Eyssen et al. compared two approaches to OT—client-centred OT based on the Canadian Practice Process Framework and usual-care OT [26]. Waliño-Paniagua et al. evaluated the additional effect of virtual reality because both groups received conventional OT [24]. Rietberg et al. evaluated a multidisciplinary programme in which OT was one of several possible rehabilitation components [29]. Esnouf et al. evaluated functional electrical stimulation against physiotherapy; an occupational therapist administered the COPM as a blinded assessor but did not provide the intervention [23].

Owing to differences in intervention content, comparator type, outcome constructs, follow-up periods, and reporting formats, a pooled effect estimate was not calculated. In accordance with the Synthesis Without Meta-Analysis reporting guideline [3], studies were grouped by outcome domain and by the directness of their evidence for OT. Between-group findings at the first post-intervention assessment were prioritized. Longer-term findings and within-group changes were reported separately and were not interpreted as evidence of comparative effectiveness (Table 2).

### 3.4. Sample Characteristics

The seven primary reports described 488 randomized participants. Randomized sample sizes ranged from 20 participants in the pilot sleep-intervention trial [25] to 269 participants in the cluster-randomized trial [26]. Because attrition and analytical approaches differed, the post-randomization samples were smaller in several studies. For example, 53 of 64 randomized participants were analyzed by Esnouf et al. [23], 44 of 48 were analyzed by Rietberg et al. [29], and 16 of 26 were analyzed by Waliño-Paniagua et al. [24].

The reported mean ages ranged from approximately 39 years in Akbarfahimi et al. [25] to 55 years in Esnouf et al. [23]. Most samples included a greater proportion of women, although Waliño-Paniagua et al. included equal numbers of women and men among the 16 completers [24].

The clinical populations were heterogeneous. Esnouf et al. included participants with secondary progressive MS and unilateral foot drop [23], whereas Waliño-Paniagua et al. included participants with relapsing–remitting MS and EDSS scores of 3.5–6.0 [24]. Kos et al. recruited ambulatory participants with a high perceived impact of fatigue [28], Rietberg et al. specifically included ambulatory adults with chronic fatigue [29], and Kessler et al. included participants with relapsing–remitting, primary progressive, and secondary progressive MS [27]. Among studies with fixed treatment schedules, intervention duration ranged from 3 to 18 weeks [23,28]. This variability limited direct comparison across trials.

### 3.5. Occupational Performance

Three studies assessed self-perceived occupational performance using the COPM [23,27,28]. Additional studies assessed disability, participation, autonomy, or manual dexterity [24,26,29].

In the waitlist-controlled trial by Kessler et al., Occupational Performance Coaching produced larger improvements compared to no concurrent intervention at 10 weeks [27]. The mean change in COPM satisfaction was 3.67 points (SD 2.27) in the coaching group and 0.70 points (SD 1.09) in the waitlist group (*p* < 0.001; Cohen’s d = 1.68). COPM performance increased by 2.73 points (SD 1.99) and 0.56 points (SD 0.67), respectively (*p* = 0.002; d = 1.49). Improvements were maintained within the original intervention group 8 weeks after coaching ended. However, because the waitlist group subsequently received the intervention, this follow-up did not provide a contemporaneous controlled estimate [27].

Kos et al. found improvements over time in both the self-management OT and relaxation groups for COPM performance (F = 13.1, *p* = 0.001) and satisfaction (F = 10.4, *p* = 0.001), but the between-group differences were not statistically significant [28]. At 3 months, 71% of participants receiving self-management OT and 27% receiving relaxation achieved a clinically relevant improvement in COPM performance. This difference in proportions suggested a possible advantage for SMOoTh, although the small sample and nonsignificant overall between-group analysis precluded a firm conclusion [28].

Esnouf et al. reported greater COPM improvement with functional electrical stimulation than with home physiotherapy [23]. The median change in performance was 1.1 points (IQR 0.1–2.0) with the stimulator and 0.0 points (IQR 0.0–0.9) with exercise (*p* = 0.038). The corresponding median changes in satisfaction were 1.7 points (IQR 0.3–2.7) and 0.0 points (IQR 0.0–1.0), respectively (*p* = 0.007). Nevertheless, the occupational therapist acted only as a blinded COPM assessor, and no OT intervention was delivered during the 18-week period. These findings therefore support an effect of functional electrical stimulation on occupation-related goals, rather than an independent effect of OT [23].

The larger trial by Eyssen et al. found no between-group differences in its primary disability, participation, or autonomy outcomes at either 4 or 8 months [26]. Process measures, including the perceived client-centeredness and information provision by therapy, favored the CPPF-based intervention, but this did not translate into superior functional outcomes [26].

Waliño-Paniagua et al. found no significant between-group differences in any PPT, JTT, or GPT variable after treatment [24]. Some individual tasks improved within each group, but these changes did not demonstrate that OT plus virtual reality was superior to conventional OT. Because both groups received the same conventional OT programme, this study estimated only the additional effect of virtual reality [24].

Rietberg et al. found no convincing between-group differences in functional independence, the impact of MS, or participation and autonomy [29]. Two isolated secondary comparisons involving DIP symptoms and IPA mobility favored multidisciplinary rehabilitation during follow-up; however, the investigators considered that these findings could have arisen through multiple testing. Moreover, the contribution of OT could not be distinguished from those of physiotherapy, social work, psychology, or other components [29].

The direction of effects on occupational performance was not consistent. One small waitlist-controlled trial showed large short-term improvements following direct OT coaching [27], whereas a direct OT trial with an active comparator showed improvement in both groups without clear comparative superiority [28]. Client-centered OT was not superior to usual-care OT [26], and adding virtual reality to conventional OT did not improve manual dexterity relative to OT alone [24]. The favorable COPM findings for functional electrical stimulation constituted indirect evidence and should not be attributed to OT [23].

### 3.6. Physical and Cognitive Fatigue

Four studies assessed fatigue using the FIS, FSS, MFIS, or CIS [25,26,28,29]. Results were not pooled because of substantial differences in interventions, comparators, fatigue constructs, follow-up periods, and reporting formats.

Akbarfahimi et al. found that adding a sleep-targeted OT intervention to usual care improved the FIS total score (*p* < 0.001; Cohen’s d = 0.82) and FSS score (*p* < 0.001; d = 0.76) [25]. Cognitive, physical, and social FIS domains also favored the intervention (d = 0.68–0.76). However, this pilot study included only 20 participants, and baseline FIS differences required comparison of gain scores; therefore, the magnitude of the effects should be interpreted cautiously [25].

Conversely, Eyssen et al. reported that physical and total fatigue scores favored usual-care OT over CPPF-based client-centered OT at 4 months, although these differences were not maintained at 8 months [26]. Kos et al. found no significant differential effects of self-management OT versus relaxation on MFIS or CIS scores [28]. Similarly, Rietberg et al. found no differences between multidisciplinary rehabilitation and MS-nurse consultation in CIS-20R total scores at 12 weeks (*p* = 0.39) or 24 weeks (*p* = 0.14), nor in CIS-20R subscale, MFIS, or FSS scores [29]. Because the intervention was multidisciplinary and the comparator included activity-planning and energy-conservation advice, the independent contribution of OT could not be established [29].

Kessler et al. was excluded from the fatigue synthesis because they administered the MSIS-29 rather than the MFIS [27]. The MSIS-29 assesses the perceived physical and psychological impact of MS on daily life rather than physical and cognitive fatigue; neither domain differed significantly between groups [27].

Overall, fatigue findings were inconsistent. One small pilot trial reported moderate-to-large effects favouring a sleep-targeted OT intervention [25], two trials found no comparative benefit [28,29], and one large trial transiently favored usual-care OT [26]. Therefore, the available evidence does not consistently demonstrate that OT interventions reduce physical or cognitive fatigue in adults with MS.

### 3.7. Certainty of Evidence

Some included RCTs reported improvements in occupational performance or fatigue-related outcomes following occupational therapy interventions; however, the direction and magnitude of the effects were inconsistent across studies. The certainty of evidence was rated as very low for both outcomes because of inconsistency, indirectness, and imprecision, with additional concerns regarding risk of bias for fatigue-related outcomes. Therefore, confidence in these findings is limited, and the true effects may differ substantially from those observed (Table 3).

### 3.8. Adverse Effects and Adherence

Adherence, attrition, and safety reporting varied substantially across studies (Supplementary Table S2) [23,24,25,26,27,28,29]. Attrition ranged from no reported losses in the small sleep-targeted OT trial [25] to 38.5% in the virtual-reality trial [24], while adherence was inconsistently quantified. Only Kos et al. explicitly reported that the intervention was well tolerated and that no adverse effects occurred [28]; most trials did not systematically assess or report adverse events [23,24,25,26,27,29]. Accordingly, the absence of reported events should not be interpreted as evidence of safety.

## 4. Discussion

### 4.1. Occupational Performance

The present synthesis did not demonstrate a consistent comparative benefit of OT across occupational performance and related activity and participation outcomes, and the certainty of evidence was very low (Table 3) [23,24,26,27,28,29]. The largest short-term effects were observed for Occupational Performance Coaching compared with a waitlist; however, this estimate originated from a small trial, and the subsequent crossover prevented controlled assessment of whether the improvements were maintained [27]. When OT was compared with an active intervention or usual-care OT, both groups improved or no clear comparative advantage emerged, as observed by Kos et al. and Eyssen et al. [26,28]. This pattern indicates that the estimated effects may depend substantially on the intensity and content of the comparator, as well as on the specific intervention evaluated [26,27,28].

Technology-based and multidisciplinary interventions require separate interpretation. The COPM improvements reported by Esnouf et al. reflected the effect of functional electrical stimulation compared with home physiotherapy, while OT was only involved in outcome assessment [23]. Similarly, Waliño-Paniagua et al. estimated the additional effect of virtual reality because both groups received conventional OT, whereas Rietberg et al. did not permit the contribution of OT to be separated from the other components of their multidisciplinary rehabilitation program [24,29]. These studies inform occupation-related outcomes or combined rehabilitation approaches but do not establish the independent effectiveness of OT [23,24,29].

### 4.2. Physical and Cognitive Fatigue

The available RCT evidence did not consistently demonstrate that OT reduces the severity or impact of fatigue, and the certainty of evidence was very low (Table 3) [25,26,28,29]. A small pilot trial reported favorable effects of a sleep-targeted OT intervention on the FIS and FSS; however, its limited sample and baseline imbalance reduced confidence in the magnitude of these findings [25]. Conversely, self-management OT did not outperform relaxation, multidisciplinary rehabilitation did not outperform MS-nurse consultation, and usual-care OT showed more favorable physical and total fatigue scores than CPPF-based client-centered OT at 4 months, although these differences were not maintained at 8 months [26,28,29].

Differences in intervention targets, comparators, instruments, and follow-up periods offer plausible explanations for the inconsistent findings [25,26,28,29]. The interventions ranged from sleep management and occupational self-management to client-centred OT and multidisciplinary rehabilitation, while active comparators included relaxation and nurse-led advice incorporating activity planning and energy conservation [25,26,28,29]. Moreover, the FIS, FSS, MFIS, and CIS assess related but nonidentical fatigue constructs, limiting direct comparison across trials [25,26,28,29]. Maintaining measurement specificity was also important because the MSIS-29 assesses the perceived impact of MS rather than fatigue and was therefore not included as a fatigue measure [27].

Rather than replicating the recent Cochrane review, the present review addresses a narrower and attribution-focused question. The Cochrane review examined a broader range of outcomes, including daily functioning, participation, health-related quality of life, and adverse effects, and included randomized and nonrandomized controlled studies, with searches conducted through November 2024 [8]. In contrast, the present review was restricted to RCTs assessing occupational performance and physical and cognitive fatigue, extended the search through to 10 August 2026, and classified each treatment contrast as direct, comparative, adjunctive, embedded, or indirect evidence. This approach enabled explicit examination of whether the observed effects could be reasonably attributed to OT itself. Yu et al. reported benefits across several activities of daily living and sleep outcomes, but their broader eligibility criteria included randomized, nonrandomized, and uncontrolled studies, limiting direct comparison with the present RCT-only synthesis [30]. Previous OT-focused reviews also described a heterogeneous and outcome-dependent evidence base for activity, participation, occupational performance, and fatigue management in people with MS [6,13,31]. Overall, selected goal-based interventions, particularly Occupational Performance Coaching, may improve self-perceived performance in the short term; however, favorable signals were confined to small trials, and the available evidence does not establish a general, sustained, or independently attributable effect across OT approaches [8,13,26,27,28,29,30].

### 4.3. Strengths, Limitations, and Future Research

This review was strengthened by its protocol registration, systematic search across seven databases, restriction to RCTs, independent study selection and data extraction, outcome-level risk-of-bias assessment, synthesis following SWiM guidance, and evaluation of evidence certainty using GRADE. Restricting eligibility to RCTs strengthened the causal interpretation of comparative effects, although it limited the evidence available on implementation and real-world feasibility.

Several limitations should be considered. Only seven RCTs met the eligibility criteria, and some had small samples [23,24,25,26,27,28,29]. Substantial differences in intervention content, comparators, outcome constructs, reporting formats, and follow-up periods precluded a defensible meta-analysis and meaningful subgroup analyses by intervention characteristics or MS phenotype [23,24,25,26,27,28,29]. Moreover, the functional electrical stimulation trial did not deliver OT as the intervention, and the multidisciplinary rehabilitation trial did not permit the independent contribution of OT to be determined, thereby introducing indirectness [23,29]. Most findings were limited to short-term follow-up, while incomplete reporting of adherence, attrition, and adverse events prevented firm conclusions regarding sustainability, acceptability, or safety [23,24,25,26,27,28,29]. Because grey literature and trial registries were not searched and eligibility was restricted to published RCTs, publication bias cannot be excluded. The focused review question also prevented comprehensive evaluation of other potentially relevant outcomes, including quality of life, mood, and broader participation.

Future studies should use adequately powered, preferably multicentre, RCTs with clearly defined OT content, providers, theoretical frameworks, co-interventions, and comparators. Trials should prespecify analyses according to MS phenotype and disability level, adopt standardized outcome sets for occupational performance and fatigue, and include follow-up assessments extending for at least 6–12 months. Treatment fidelity, adherence, attrition and its reasons, adverse events, and resource use should be systematically reported. These improvements are required before firm conclusions can be drawn regarding the effectiveness, durability, safety, and cost-effectiveness of OT interventions for adults with MS.

### 4.4. Clinical and Practical Implications

Given the very low certainty of evidence for both occupational performance and fatigue outcomes, the findings support cautious, individualized application of OT rather than a general conclusion of effectiveness (Table 3) [23,24,25,26,27,28,29]. Occupational Performance Coaching produced large short-term improvements in COPM scores compared with a waitlist, whereas self-management OT did not clearly outperform relaxation, and CPPF-based client-centred OT was not superior to usual-care OT [26,27,28]. Individualized goal setting and COPM-based monitoring may therefore be clinically useful, although expected benefits should be interpreted considering the small samples, heterogeneous interventions, and limited comparative evidence [23,27,28].

Fatigue management should likewise be tailored to each patient’s occupational priorities because a small sleep-targeted OT trial reported improvement, while other trials found no consistent benefit or transiently favored usual care [25,26,28,29]. Adjunctive virtual reality did not improve manual dexterity beyond conventional OT, while the effects of functional electrical stimulation and multidisciplinary rehabilitation could not be independently attributed to OT [23,24,29]. Clinical implementation should therefore prioritize clearly defined occupation-focused components, patient-centered goals, and systematic monitoring of outcomes, adherence, attrition, and adverse events; stronger recommendations require adequately powered RCTs with standardized interventions and longer follow-up [23,24,25,26,27,28,29].

## 5. Conclusions

The evidence from seven RCTs does not support a consistent effect of OT on occupational performance or physical and cognitive fatigue in adults with MS. Although a small waitlist-controlled trial of Occupational Performance Coaching and a pilot sleep-targeted OT trial reported favorable short-term effects, these signals were not consistently reproduced against active or usual-care comparators. Moreover, adjunctive technology-assisted and embedded multidisciplinary interventions did not permit attribution of effects to OT itself. The certainty of evidence was very low because of inconsistency, indirectness, and imprecision, with additional risk-of-bias concerns for fatigue outcomes; therefore, the findings remain preliminary. Adequately powered RCTs should isolate clearly defined OT components, use appropriate comparators and standardized outcomes, include longer follow-up, and systematically report adherence and adverse events before conclusions about effectiveness and sustainability can be considered robust.

## Figures and Tables

**Table 1:** Selection criteria used in the systematic review.

Category	Inclusion	Exclusion
Population	Adults aged ≥18 years with a clinical diagnosis of MS. Any MS phenotype, disability severity, disease duration, clinical status, treatment status, or rehabilitation setting was eligible.	Participants aged <18 years; diagnoses other than MS; or mixed samples for which data from adults with MS could not be extracted.
Intervention	Direct or comparative OT interventions, or adjunctive, embedded, or indirect rehabilitation approaches relevant to OT practice, lasting ≥3 weeks.	Interventions without an identifiable relationship to OT practice, occupational goals, or an OT component.
Comparison	Active comparators, usual care, alternative rehabilitation interventions, waitlist controls, or no concurrent intervention.	Absence of a randomized comparison group.
Outcomes	At least one validated measure of occupational performance or related activity, participation, autonomy, disability, or manual-dexterity outcomes; or physical or cognitive fatigue severity or impact.	Absence of an eligible outcome or insufficient baseline or post-intervention data.
Study design	Randomized controlled trials, with pre- and post-assessment.	Quasi-randomized, non-randomized, observational, single-group, retrospective, prospective cohort, cross-sectional, qualitative, or case studies.

**Table 2:** Characteristics and principal findings of the included randomized controlled trials.

Study, Country, and Design	Sample, Setting, and MS Characteristics	Intervention Versus Comparator	Dose and Assessment Timepoints	Outcomes and Main Between-Group Findings
[23]; UK; parallel RCT	Hospital; SPMS with unilateral foot drop; EDSS: 4.0–6.5; mean age: 53/57 years. Randomized: 64 (32/32); analyzed: 53 (26/27).	**I:** Daily ODFS during mobility; no OT intervention (OT only administered the blinded COPM assessment). **C:** Individualized home physiotherapy (~30 min once or twice daily).	18 weeks; baseline and week 18.	Median COPM change favored ODFS: performance 1.1 vs. 0.0 (*p* = 0.038); satisfaction 1.7 vs. 0.0 (*p* = 0.007). Indirect evidence for OT.
[24]; ES; assessor-blinded parallel RCT	Community; RRMS; EDSS eligibility: 3.5–6.0; mean age: 46.8/46.1 years. Randomized: 26; analyzed: 16 (8/8).	**I:** Conventional upper-limb OT plus 20-min game-based VR; OT-delivered. **C:** The same 30-min conventional OT without VR.	20 sessions/10 weeks (2/week); pre/post only.	Manual dexterity (PPT, JTT, GPT): no between-group differences (all *p* > 0.05). This estimates the incremental effect of VR, not OT versus no OT.
[25]; IR; assessor-blinded pilot RCT	OT clinic; MS with poor sleep (PSQI >= 5); EDSS < 6; phenotype: NR; mean age: 37.5/39.7 years. Randomized/analyzed: 20 (10/10).	**I:** Usual-care OT plus OT-delivered, PEOP-based sleep program. **C:** Usual-care OT (motor, cognitive, ADL, and assistive-device training).	2–3 sessions/8 weeks (30–45 min); baseline and 8 weeks.	Gain scores favored intervention for FIS total (*p* < 0.001; d = 0.82) and FSS (*p* < 0.001; d = 0.76). Small pilot with baseline FIS imbalance.
[26]; NL; multicenter cluster RCT	Thirteen outpatient centers; mixed phenotypes; >1 ADL limitation; EDSS: 5.0/5.1; mean age: 44.4/47.1 years. ITT: 269 (156/113).	**I:** Individualized, client-centered OT structured by the CPPF. **C:** Individualized usual-care OT.	Individualized dose; baseline, 4 months, and 8 months.	No differences in disability, participation, or autonomy at 4 or 8 months. Fatigue favored usual-care OT at 4 months only.
[27]; CA; waitlist-controlled RCT	Community; RRMS/PPMS/SPMS: 11/2/2 vs. 10/2/2; EDSS NR; mean age: 44.1/49.9 years. Randomized: 30 (15/15); baseline: 29; 10-week analysis: 23 (11/12).	**I:** Telephone Occupational Performance Coaching. **C:** No-contact waitlist for 10 weeks, followed by coaching.	6 sessions/10 weeks; baseline, 10 weeks, and 20 weeks.	At 10 weeks, mean COPM change favored coaching: satisfaction 3.67 vs. 0.70 (*p* < 0.001; d = 1.68); performance 2.73 vs. 0.56 (*p* = 0.002; d = 1.49). No concurrent control after crossover.
[28]; BE; assessor-blinded parallel RCT	MS centers; definite MS; VAS fatigue >= 60; EDSS <= 5; no relapse in 3 months; phenotype NR; mean age: 37/44 years. Randomized/ITT: 31 (17/14); completed: 25 (14/11).	**I:** Individual, OT-delivered self-management program. **C:** Matched stress-management and relaxation program.	3 weekly sessions (60–90 min); baseline, post-treatment, and 3 months.	No group-by-time effects for COPM performance (*p* = 0.23), satisfaction (*p* = 0.39), or MFIS (*p* = 0.27). Clinically relevant COPM performance improvement at 3 months: 71% vs. 27% (exploratory).
[29]; NL; single-blind parallel RCT	Outpatient; ambulatory adults with chronic MS fatigue; RRMS/SPMS/PPMS: 16/5/2 vs. 12/7/6; median EDSS: 3/4; mean age: 45/47 years. Randomized: 48 (23/25); analyzed: 44 (21/23).	**I:** Individualized multidisciplinary rehabilitation including OT, physiotherapy, and/or social work after team assessment. **C:** MS-nurse consultation with activity-planning and energy-conservation advice.	12 weeks; baseline, 12 weeks, and 24 weeks.	No CIS-20R difference at 12 (*p* = 0.39) or 24 weeks (*p* = 0.14), and no MFIS/FSS differences. Isolated secondary findings may reflect multiplicity; the OT effect was not isolable.

Group values are presented as intervention/comparator unless otherwise indicated; between-group findings are prioritized. ADL: activities of daily living; BE: Belgium; C: comparator; CA: Canada; CIS-20R: Checklist Individual Strength; COPM: Canadian Occupational Performance Measure; CPPF: Canadian Practice Process Framework; d: Cohen’s d; EDSS: Expanded Disability Status Scale; ES: Spain; FIS: Fatigue Impact Scale; FSS: Fatigue Severity Scale; GPT: Grooved Pegboard Test; I: intervention; IR: Iran; ITT: intention-to-treat; JTT: Jebsen–Taylor Hand Function Test; MFIS: Modified Fatigue Impact Scale; MS: multiple sclerosis; NL: the Netherlands; NR: not reported; ODFS: Odstock Dropped Foot Stimulator; OT: occupational therapy; PEOP: Person–Environment–Occupation–Performance model; PPMS: primary progressive multiple sclerosis; PPT: Purdue Pegboard Test; PSQI: Pittsburgh Sleep Quality Index; RCT: randomized controlled trial; RRMS: relapsing-remitting multiple sclerosis; SPMS: secondary progressive multiple sclerosis; UK: United Kingdom; VAS: visual analog scale; VR: virtual reality.

**Table 3:** GRADE certainty-of-evidence assessment for the principal outcomes.

Certainty of Evidence							N° of Patients		Effect		Certainty	Importance
N° of Studies	Study Design	Risk of Bias	Inconsistency	Indirectness	Imprecision	Other Considerations	[Intervention]	[Comparison]	Relative (95% CI)	Absolute (95% CI)		
**Occupational performance**												
3	RCT	Not serious	Serious	Serious	Serious	None	51	50	Not estimable		Very low	Important
**Impact of fatigue on occupational performance**												
4	RCT	Serious	Serious	Serious	Serious	None	201	157	Not estimable		Very low	Important

CI: Confidence Interval; RCT: randomized controlled trial.

## Data Availability

The datasets generated or used during this research are available from the corresponding author upon reasonable request.

## Supplementary Materials

The following supporting information can be downloaded at https://www.mdpi.com/article/10.3390/jcm15208060/s1, Table S1: PRISMA 2020 Checklist; Supplementary Material S1: Complete database search strategies and results; Table S2: Adherence, attrition, and safety reporting in the included randomized controlled trials.
