# Supplementary material for: Effects of Occupational Therapy Interventions on Occupational Performance and Physical and Cognitive Fatigue in Adults with Multiple Sclerosis: A Systematic Review of Randomized Controlled Trials
Source: J Clin Med. 2026 Oct 17;15(20):8060. doi: 10.3390/jcm15208060 (PMC13706231; doi:10.3390/jcm15208060)
Supplement: Supplementary file 1 [file jcm-15-08060-s001.zip › Suplementary Material S1. Complete Electronic Search Strategies.pdf]

Supplementary Material S1

Complete Electronic Search Strategies

Associated manuscript

Effects of Occupational Therapy Intervention on Occupational Performance and Physical and Cognitive Fatigue in Adults with Multiple Sclerosis: A Systematic Review of Randomized Controlled Trials

Search overview

All databases were searched from inception to 10 August 2026. No restrictions on language or publication date were applied, and no study-design filter was used. The PubMed strategy was used as the master strategy and adapted to the syntax and searchable fields of each database. Grey-literature sources were not searched. The searches yielded 3,734 records before deduplication, as reported in Figure 1 of the manuscript.

1. MEDLINE

Platform: PubMed **Search date:** 10 August 2026 **Records retrieved:** n = 954

Search strategy

("multiple sclerosis" OR "disseminated sclerosis" OR "MS" OR "relapsing-remitting multiple sclerosis" OR "RRMS" OR "secondary progressive multiple sclerosis" OR "SPMS" OR "primary progressive multiple sclerosis" OR "PPMS" OR "progressive-relapsing multiple sclerosis" OR "clinically isolated syndrome" OR "CIS" OR "benign multiple sclerosis" OR "malignant multiple sclerosis") AND ("occupational therapy" OR "occupational rehabilitation" OR "occupational intervention" OR "occupational treatment" OR "OT") AND ("adult" OR "adults" OR "middle aged" OR "young adult" OR "young adults" OR "adult population")

2. Scopus

Platform: Elsevier Scopus **Search date:** 10 August 2026 **Records retrieved:** n = 1973

Search strategy

TITLE-ABS-KEY("multiple sclerosis" OR "disseminated sclerosis" OR "MS" OR "relapsing-remitting multiple sclerosis" OR "RRMS" OR "secondary progressive multiple sclerosis" OR "SPMS" OR "primary progressive multiple sclerosis" OR "PPMS" OR "progressive-relapsing multiple sclerosis" OR "clinically isolated syndrome" OR "CIS" OR "benign multiple sclerosis" OR "malignant multiple sclerosis") AND TITLE-ABS-KEY("occupational therapy" OR "occupational rehabilitation" OR "occupational intervention" OR "occupational treatment" OR "OT") AND TITLE-ABS-KEY("adult" OR "adults" OR "middle aged" OR "young adult" OR "young adults" OR "adult population")

3. Cochrane Central Register of Controlled Trials (CENTRAL)

Platform: Cochrane Library **Search date:** 10 August 2026 **Records retrieved:** n = 64

Database segment: Trials (CENTRAL)

Search strategy

#1 MeSH descriptor: [Multiple Sclerosis] explode all trees
#2 ("multiple sclerosis" OR "disseminated sclerosis" OR "MS" OR "relapsing-remitting multiple sclerosis" OR "RRMS" OR "secondary progressive multiple sclerosis" OR "SPMS" OR "primary progressive multiple sclerosis" OR "PPMS" OR "progressive-relapsing multiple sclerosis" OR "clinically isolated syndrome" OR "CIS" OR "benign multiple sclerosis" OR "malignant multiple sclerosis"):ti,ab,kw
#3 #1 OR #2
#4 MeSH descriptor: [Occupational Therapy] explode all trees
#5 ("occupational therapy" OR "occupational rehabilitation" OR "occupational intervention" OR "occupational treatment" OR "OT"):ti,ab,kw
#6 #4 OR #5
#7 MeSH descriptor: [Adult] explode all trees
#8 ("adult" OR "adults" OR "middle aged" OR "young adult" OR "young adults" OR "adult population"):ti,ab,kw
#9 #7 OR #8
#10 #3 AND #6 AND #9

4. Web of Science Core Collection

Platform: Clarivate Web of Science Search **date:** 10 August 2026 **Records retrieved:** n = 345

Search strategy

TS=("multiple sclerosis" OR "disseminated sclerosis" OR "MS" OR "relapsing-remitting multiple sclerosis" OR "RRMS" OR "secondary progressive multiple sclerosis" OR "SPMS" OR "primary progressive multiple sclerosis" OR "PPMS" OR "progressive-relapsing multiple sclerosis" OR "clinically isolated syndrome" OR "CIS" OR "benign multiple sclerosis" OR "malignant multiple sclerosis") AND TS=("occupational therapy" OR "occupational rehabilitation" OR "occupational intervention" OR "occupational treatment" OR "OT") AND TS=("adult" OR "adults" OR "middle aged" OR "young adult" OR "young adults" OR "adult population")

5. APA PsycINFO

Platform: EBSCOhost **Search date:** 10 August 2026 **Records retrieved:** n = 232

Search strategy

(DE "Multiple Sclerosis" OR TI ("multiple sclerosis" OR "disseminated sclerosis" OR "MS" OR "relapsing-remitting multiple sclerosis" OR "RRMS" OR "secondary progressive multiple sclerosis" OR "SPMS" OR "primary progressive multiple sclerosis" OR "PPMS" OR "progressive-relapsing multiple sclerosis" OR "clinically isolated syndrome" OR "CIS" OR "benign multiple sclerosis" OR "malignant multiple sclerosis") OR AB ("multiple sclerosis" OR "disseminated sclerosis" OR "MS" OR "relapsing-remitting multiple sclerosis" OR "RRMS" OR "secondary progressive multiple sclerosis" OR "SPMS" OR "primary progressive multiple sclerosis" OR "PPMS" OR "progressive-relapsing multiple sclerosis" OR "clinically isolated syndrome" OR "CIS" OR "benign multiple sclerosis" OR "malignant multiple sclerosis")) AND (DE "Occupational Therapy" OR TI

("occupational therapy" OR "occupational rehabilitation" OR "occupational intervention" OR "occupational treatment" OR "OT") OR AB ("occupational therapy" OR "occupational rehabilitation" OR "occupational intervention" OR "occupational treatment" OR "OT")) AND (TI ("adult" OR "adults" OR "middle aged" OR "young adult" OR "young adults" OR "adult population") OR AB ("adult" OR "adults" OR "middle aged" OR "young adult" OR "young adults" OR "adult population"))

6. CINAHL Complete

Platform: EBSCOhost **Search date:** 10 August 2026 **Records retrieved:** n = 45

Search strategy

(MH "Multiple Sclerosis+" OR TI ("multiple sclerosis" OR "disseminated sclerosis" OR "MS" OR "relapsing-remitting multiple sclerosis" OR "RRMS" OR "secondary progressive multiple sclerosis" OR "SPMS" OR "primary progressive multiple sclerosis" OR "PPMS" OR "progressive-relapsing multiple sclerosis" OR "clinically isolated syndrome" OR "CIS" OR "benign multiple sclerosis" OR "malignant multiple sclerosis") OR AB ("multiple sclerosis" OR "disseminated sclerosis" OR "MS" OR "relapsing-remitting multiple sclerosis" OR "RRMS" OR "secondary progressive multiple sclerosis" OR "SPMS" OR "primary progressive multiple sclerosis" OR "PPMS" OR "progressive-relapsing multiple sclerosis" OR "clinically isolated syndrome" OR "CIS" OR "benign multiple sclerosis" OR "malignant multiple sclerosis")) AND (MH "Occupational Therapy+" OR TI ("occupational therapy" OR "occupational rehabilitation" OR "occupational intervention" OR "occupational treatment" OR "OT") OR AB ("occupational therapy" OR "occupational rehabilitation" OR "occupational intervention" OR "occupational treatment" OR "OT")) AND (MH "Adult+" OR TI ("adult" OR "adults" OR "middle aged" OR "young adult" OR "young adults" OR "adult population") OR AB ("adult" OR "adults" OR "middle aged" OR "young adult" OR "young adults" OR "adult population"))

7. ProQuest Central

Platform: ProQuest Search **date:** 10 August 2026 **Records retrieved:** n = 121

Search strategy

NOFT("multiple sclerosis" OR "disseminated sclerosis" OR "MS" OR "relapsing-remitting multiple sclerosis" OR "RRMS" OR "secondary progressive multiple sclerosis" OR "SPMS" OR "primary progressive multiple sclerosis" OR "PPMS" OR "progressive-relapsing multiple sclerosis" OR "clinically isolated syndrome" OR "CIS" OR "benign multiple sclerosis" OR "malignant multiple sclerosis") AND NOFT("occupational therapy" OR "occupational rehabilitation" OR "occupational intervention" OR "occupational treatment" OR "OT") AND NOFT("adult" OR "adults" OR "middle aged" OR "young adult" OR "young adults" OR "adult population")

Record-count note

The database-specific record counts reported above were obtained from the final searches conducted on 10 August 2026 and correspond to the totals presented in Figure 1.
