# Supplementary material for: Effects of Occupational Therapy Interventions on Occupational Performance and Physical and Cognitive Fatigue in Adults with Multiple Sclerosis: A Systematic Review of Randomized Controlled Trials
Source: J Clin Med. 2026 Oct 17;15(20):8060. doi: 10.3390/jcm15208060 (PMC13706231; doi:10.3390/jcm15208060)
Supplement: Supplementary file 1 [file jcm-15-08060-s001.zip › Supplementary Table S2. Adherence Attrition Safety.pdf]

Supplementary Table S2. Adherence, attrition, and safety reporting in the included randomized controlled trials

Study	Intervention / comparator	Randomized, n (I/C)	Completed / analysed, n (I/C)	Attrition and reasons	Adherence / attendance	Adverse events / safety
**Esnouf et al. (2010)**	ODFS/FES vs home physiotherapy	64 (32/32)	53 (26/27)	I: 6/32 (18.8%): 1 stopped before treatment (light-headedness on FES application), 1 lost to follow-up, and 4 discontinued (femur fracture after a fall; swollen legs; device inconvenience; worsening MS/unable to attend). C: 5/32 (15.6%): 2 lost to follow-up, 2 discontinued (fall; low-back pain; both reported unrelated), and 1 was excluded for protocol breach.	Eighteen-week programme. Quantitative device-use and exercise adherence were NR; reassessment was completed by 26/32 and 27/32.	No formal aggregate AE analysis. Light-headedness occurred during initial FES application. The exercise-group fall and low-back pain were explicitly reported as unrelated; causal attribution for other discontinuation events was NR.
**Waliño-Paniagua et al. (2019)**	Conventional OT + game-based VR video-capture training vs conventional OT	26 (arm allocation NR)	16 (8/8)	10/26 (38.5%) did not complete the study because of MS relapses or noncompliance; the reasons were not allocated by arm.	Twenty sessions were planned (2/week). Attendance percentage was NR; noncompliance contributed to attrition.	No systematic AE reporting. Relapses occurred among noncompleters; arm and intervention relatedness were NR.
**Akbarfahimi et al. (2020)**	Usual OT + sleep-targeted programme vs usual OT	20 (10/10)	20 (10/10)	No attrition reported; all randomized participants appeared in the pre/post analyses.	Eight weeks; 2–3 sessions/week (30–45 min) with telephone follow-up. Quantitative attendance/compliance was NR.	AEs and safety-monitoring methods were NR.
**Eyssen et al. (2013)**	CPPF-based client-centred OT vs usual-care OT	269 (156/113)	ITT: 269 (156/113) Completed: ≈255 (148/107) *	Reported dropout was 5.1% I and 5.3% C (≈8 and ≈6 participants; ≈14/269 overall). Reasons were NR. Twelve treatment diaries were missing.	Therapy diaries were available for 257/269. Overall intervention adherence was described as successful, but participant-level compliance was not quantified. Mean sessions: 7.7 (SD 6.7) vs 11.9 (SD 7.8).	AEs and safety-monitoring methods were NR.
**Kessler et al. (2024)**	OPC vs waitlist (then crossover)	30 (15/15)	T2: 23 (11/12) Intervention completed: 10/15 I; 8/12 crossover C	Seven of 30 (23.3%; 4 I/3 C) were not included at T2. Across study/intervention phases, withdrawal reasons were coaching not needed/receiving psychotherapy (n=3), disliked telephone coaching (n=2), illness (n=2), preferred exercise therapy (n=1), severe disability/frustration (n=1), and no reason (n=1). One C participant withdrew after baseline and requested data deletion.	Attended ≥4 sessions: 12 I and 8 C after crossover; completed intervention: 10 I and 8 C. Mean session duration 34 min (range 8–57). Fidelity reached the ≥80% target after retraining.	No formal AE reporting. Illness and increased MS symptoms appeared among withdrawal reasons; causal attribution was NR.
**Kos et al. (2016)**	SMOoTh self-management OT vs relaxation	31 (17/14)	Compliers: 25 (14/11) ITT: 31 (17/14)	I: 3/17 (18%); C: 3/14 (21%). Reasons were not fully itemized; two dropouts were hospitalized (arm and causal attribution NR).	Three weekly 60–90-min sessions in both arms. Completers: 14/17 and 11/14; session-level attendance was NR.	The programme was reported as well tolerated; no adverse effects were reported.
**Rietberg et al. (2014)**	MDR vs MS-nurse consultation	48 (23/25)	44 (21/23)	4/48 (8.3%): 2/23 I (8.7%) and 2/25 C (8.0%). Reasons were lack of motivation (n=2) and MS exacerbation (n=2); losses were evenly divided, but arm-specific reason mapping was NR.	Mean treatment time: 280 (SD 187) vs 163 (SD 106) min. Homework was assigned to 67% vs 87%; reported homework completion was 96% vs 89%.	No formal AE summary. Two withdrawals were attributed to MS exacerbation; causal relationship to the interventions was NR.

Abbreviations: AE, adverse event; C, comparator; CPPF, Canadian Practice Process Framework; FES, functional electrical stimulation; I, intervention; ITT, intention-to-treat; MDR, multidisciplinary rehabilitation programme; MS, multiple sclerosis; NR, not reported; ODFS, Odstock Foot Drop Stimulator; OPC, Occupational Performance Coaching; OT, occupational therapy; SMOoTh, self-management occupational therapy; T2, 10-week assessment; VR, virtual reality.
